# Changes in Circadian Rhythm in Chronically‐Starved Mice Are Associated With Glial Cell Density Reduction in the Suprachiasmatic Nucleus

**DOI:** 10.1002/eat.24379

**Published:** 2025-01-21

**Authors:** Annelie Zimmermann, Julia Priebe, Hanna Rupprecht, Stephan Lang, Fabienne Haberland, Katharina Schuster, Anna Staffeld, Christoph Berger, Hang Zhu, Alexander Dück, Michael Kölch, Linda Frintrop

**Affiliations:** ^1^ Institute of Anatomy Rostock University Medical Center Rostock Germany; ^2^ Department of Psychiatry, Neurology, Psychosomatics, and Psychotherapy in Childhood and Adolescence Rostock University Medical Center Rostock Germany; ^3^ German Center for Child and Adolescent Health (DZKJ) partner site Greifswald/Rostock Rostock Germany

**Keywords:** anorexia nervosa, circadian rhythm, glial cells, running wheel activity, starvation‐induced hyperactivity

## Abstract

**Objective:**

Anorexia nervosa (AN) is an eating disorder characterized by severe weight loss and associated with hyperactivity and circadian rhythm disruption. However, the cellular basis of circadian rhythm disruption is poorly understood. Glial cells in the suprachiasmatic nucleus (SCN), the principal circadian pacemaker, are involved in regulating circadian rhythms. We hypothesize that the circadian rhythm disruption in AN patients is associated with glial cell changes in the SCN.

**Method:**

In the starvation‐induced hyperactivity mouse model, mice had free access to a running wheel and received a restricted amount of food once a day, until a 25% body weight loss was reached and maintained their weight loss for two weeks. This was followed by a refeeding phase. Different daily periods of running wheel activity were defined, such as food anticipatory activity up to 4 h before feeding. Circadian rhythmicity was analyzed using the cosinor method. Gene expression was evaluated using real‐time polymerase chain reaction. Immunohistochemistry was used to quantify astrocytes, microglia, and oligodendrocytes.

**Results:**

Starvation induced changes in circadian rhythm, as indicated by changes in cosinor‐based characteristics. Refeeding reversed these effects. Additionally, there was an increase in *cryptochrome circadian regulator 1* expression and a decrease in the density of astrocytes and oligodendrocytes in the SCN after chronic starvation.

**Discussion:**

Starvation‐induced alterations in circadian rhythms are associated with molecular, and cellular changes in the hypothalamus. Reduced astrocytes and oligodendrocytes in the SCN in a mouse model of AN suggest that glial pathophysiology may play a role in circadian rhythm disruption.

**Summary**

- Patients with anorexia nervosa (AN) often suffer from circadian rhythm disruption. However, less is known about the pathophysiological mechanisms underlying this disruption.
- Using a mouse model that mimics AN, this study investigated how starvation affects circadian rhythm by focusing on glial cells.
- The findings showed that starvation alters circadian rhythms and reduces glial cells in the hypothalamus, highlighting potential mechanisms underlying circadian disruption in AN.

## 1 Introduction

Anorexia nervosa (AN) is an eating disorder in humans associated with severe weight loss, body image disturbance, and intense fear of gaining weight (Moskowitz and Weiselberg 2017). This psychiatric disorder is often a chronic disease with increasing incidence and increased mortality in adolescents in comparison to age‐matched and sex‐matched people in the general population (Herpertz‐Dahlmann 2015; van Eeden, van Hoeken, and Hoek 2021). In addition, AN is associated with hyperactivity, amenorrhea, brain volume atrophy, and hypoleptinemia (Herpertz‐Dahlmann 2015; Seitz et al. 2014). In the advanced stages of the psychiatric disorder, hypoleptinemia seemed to be linked with hyperactivity (Holtkamp et al. 2006). Furthermore, AN patients suffer from circadian rhythm disruptions, including disruptions of sleep–wake cycles, such as fragmented sleep patterns and early morning awakenings (Lauer and Krieg 2004; Lacey et al. 1975). These disruptions may also affect clinical outcomes, as indicated by the association between insomnia and poorer treatment efficacy (Allison, Spaeth, and Hopkins 2016). Nonetheless, the connection between the underlying pathophysiology of AN and circadian rhythm disruption is still not well understood.

The principal circadian pacemaker is the suprachiasmatic nucleus (SCN) of the hypothalamus, synchronizing the circadian rhythm of the daily light–dark cycles (Hastings, Maywood, and Brancaccio 2018). However, other oscillators may also play a role in circadian rhythm (Mistlberger 2009). The circadian rhythm is regulated by the protein circadian locomotor output cycles kaput (CLOCK) and its binding partner brain and muscle ARNT‐like 1 (BMAL1) (Figure S1). These proteins activate the transcription of Period (*Per1*, *Per2*, and *Per3*) and Cryptochrome (*Cry1*, *Cry2*) genes (reviewed in Cox and Takahashi 2019). Once PER and CRY proteins accumulate, they form complexes that inhibit CLOCK and BMAL1, thereby repressing their own gene transcription in a negative feedback loop. In addition to this primary feedback mechanism, a second major transcriptional mechanism involves nuclear receptors (REV‐ERBα and REV‐ERBβ) that negatively regulate the transcription of *Bmal1*.

Leptin is a hormone that is primarily synthesized in adipocytes and can reach the hypothalamus, in which cells express leptin receptors (Hebebrand, Denecke, and Antel 2023). The presence of leptin in the hypothalamus leads to a decrease in appetite, subsequently reducing food intake (Friedman 2019; Friedman and Halaas 1998). Further, leptin is known to trigger signaling pathways in the hypothalamus regulating the rhythms of clock genes (Ansarin et al. 2023). At the cellular level, glial cells are involved in the regulation of circadian rhythms (Frintrop et al. 2021; García‐Cáceres et al. 2019; Brécier et al. 2023). Astrocytes and clock neurons are integral to the circadian rhythm of the SCN (Costa and Montagnese 2021). Furthermore, studies indicate that astrocyte‐neuron interactions are a crucial component of the SCN circadian oscillator (Hastings, Maywood, and Brancaccio 2018; Hastings et al. 2023). Further studies suggest that microglia are also involved in the regulation of circadian rhythms (Sominsky et al. 2021), while the role of oligodendrocytes is unknown (Brécier et al. 2023). Thus, glial dysfunction during starvation may play a role in the circadian rhythm and sleep‐related activity changes.

To investigate potential cellular mechanisms underlying circadian rhythm disruption in AN, the starvation‐induced hyperactivity (SIH) model was used. In this model, mice received limited food until they lost 25% of their body weight and had access to running wheels. Starvation led to a phenomenon known as food anticipatory activity (FAA), which is characterized by an increase in running wheel activity prior to feeding periods (Gabloffsky et al. 2022). SIH mice developed circadian rhythm disruptions, including increased activity during the light phase (Gabloffsky et al. 2022; Beeler et al. 2021; Lewis and Brett 2010). A further study demonstrated that SIH mice exhibited altered circadian clock gene expression in peripheral tissues, including the liver, colon, and ileum, as well as in the SCN (Salaün et al. 2024). Further, we demonstrated that starvation leads to decreased glial fibrillary acidic protein‐positive (GFAP^+^) astrocyte density in the corpus callosum (CC) and cerebral cortex (Frintrop, Liesbrock, et al. 2018; Frintrop et al. 2019). Similarly, other studies have observed reduced astrocyte density in the CC, prefrontal cortex, and hippocampus in a dehydration‐induced anorexia (DIA) rat model (Reyes‐Haro et al. 2015, 2016). In this model, an increased density of microglia was found in the hippocampus and prefrontal cortex, indicating microgliosis (Reyes‐Ortega et al. 2020; Ragu‐Varman et al. 2019). However, whether glial cell changes also occur in the SCN remains unknown.

Here, we investigate whether chronic starvation leads to an increase of FAA and changes in circadian rhythm. Furthermore, we investigate whether refeeding can normalize these effects in the SIH model. In addition, the study aims to investigate whether changes in circadian rhythm are associated with endocrinological, molecular, and cellular changes in the hypothalamus.

## 2 Material and Methods

### 2.1 Animals

Female C57BL/6J mice were acquired from Janvier Labs (Le Genest‐Saint‐Isle, France). Based on the higher prevalence of AN in female patients than in male patients (Galmiche et al. 2019; Jagielska and Kacperska 2017; Keski‐Rahkonen and Mustelin 2016; Silén and Keski‐Rahkonen 2022), our study was performed in female mice only. All mice were housed individually in a cage under a 12/12 h light/dark cycle (lights on at 6 a.m.) at a temperature of 22°C ± 2°C, with unlimited access to a running wheel. Cages were changed weekly, and microbiological monitoring was done according to the Federation of European Laboratory Animal Science Associations (FELASA) recommendations. The animal studies were approved by the Review Boards for the Care of Animal Subjects of the district government of Mecklenburg‐Western Pomerania (reference number 7221.3‐1‐005/21).

### 2.2 Study Design

**FIGURE 1:**
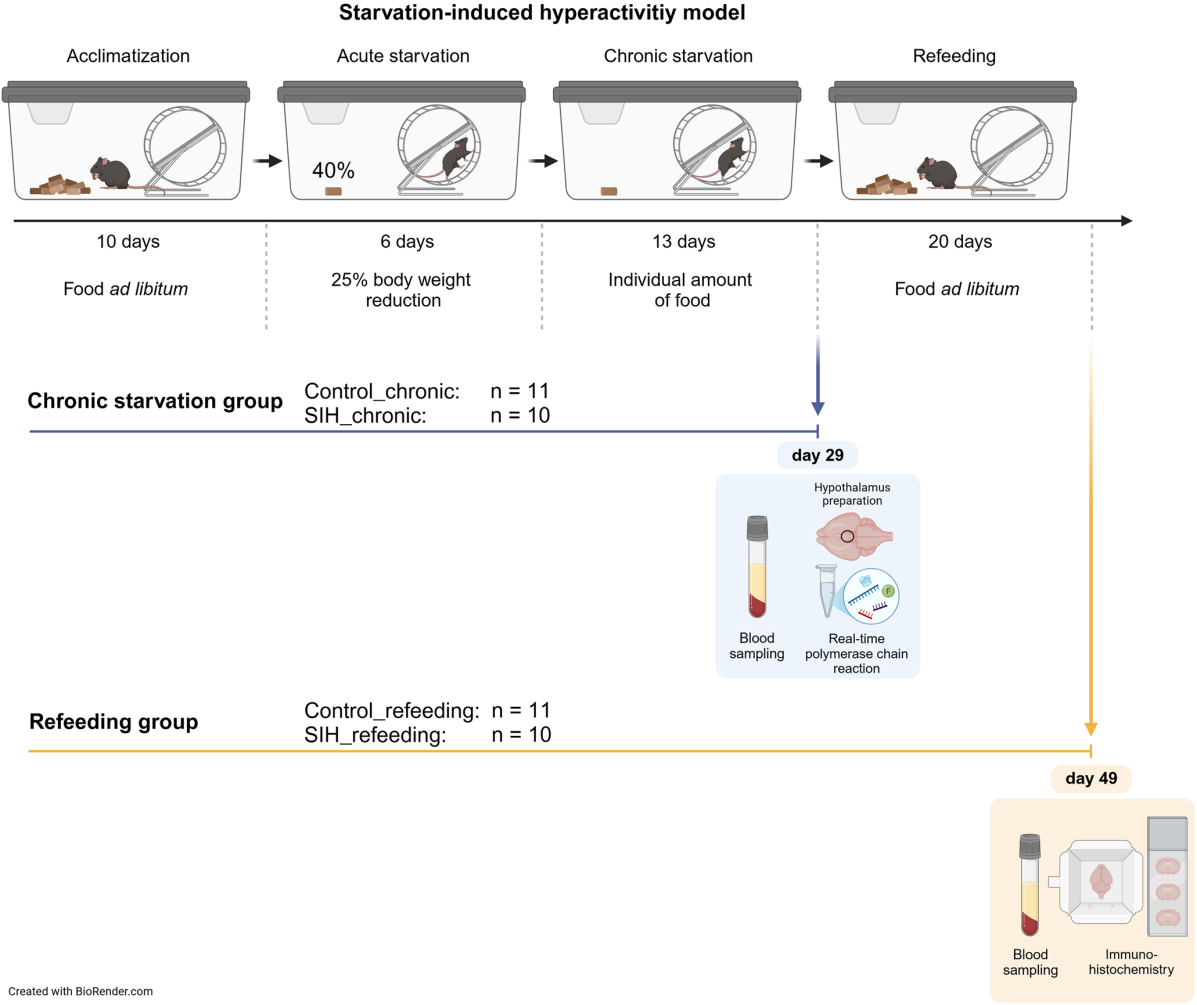
Schematic summary of the experimental set‐up including the chronic starvation and refeeding groups used in this study. Each sample collection started at nighttime and ended before feeding time (7 a.m.–1 p.m.). Created with BioRender.com.

Starvation was induced according to the SIH model, also known as the modified activity‐based anorexia (ABA) model (Frintrop, Trinh et al. 2018). The procedure of the SIH model was previously described by Frintrop, Trinh et al. (2018) and Staffeld et al. (2023). Figure 1 presents a schematic structure including the chronic starvation and refeeding groups used in the present study. At the beginning of the experiment, the mice followed a 10‐day acclimatization phase with ad libitum access to food and water. Body weight, food intake, and estrous cycle were monitored daily at 1 p.m. In addition, during the starvation phase, the mice were fed at this specific time and consumed their restricted amount of food within the 1 h feeding period (1–2 p.m.). After acclimatization, the mice were randomly assigned to different groups (Control_chronic: *n* = 11; SIH_chronic: *n* = 10; Control_refeeding: *n* = 11; SIH_refeeding: *n* = 10; four weeks old referred to as early adolescent; one animal from each SIH group died due to high vulnerability to the model). SIH mice received 40% of the baseline food intake, calculated from the food intake during the acclimatization phase until a 25% body weight reduction was reached (one‐week phase defined as acute starvation). The daily food consumption during the acclimatization phase was averaged to calculate the baseline food intake per mouse. After the target weight was reached, food intake was adjusted daily to maintain the 25% body weight reduction for another two weeks to mimic chronic starvation, ranging between 45% and 70% of the baseline food intake. During both phases, SIH mice had free access to their calculated amount of food. This was followed by a three‐week refeeding phase, during which the animals had ad libitum access to food. Control mice were fed ad libitum throughout the whole experiment. Running wheel activity was analyzed as previously described (Gabloffsky et al. 2022; Staffeld et al. 2023) (further details: Data S1). Experimental animals were removed from the study if they experienced an additional 10% weight loss within 24 h, and exhibited cramps, paralysis, abnormal breathing sounds, signs of pain, or labored breathing.

### 2.3 Circadian Rhythm Analyses

For quantitative evaluation of circadian rhythmicity the cosinor method was used by fitting the running wheel activity data with a (co)sine curve with a period T of 24 h: $Y\left(t\right)=M+A\times \cos\left(\frac{2\pi }{T}\times t+\varphi \right)$ (Cornelissen 2014; Nelson et al. 1979). The cosinor function consists of three components describing the rhythmicity: MESOR (M, midline estimating statistic of rhythm) is the mean of the measured activity and the amplitude A is the measure of the extent of the oscillation within the daily cycle. The acrophase *ϕ* represents the timing of the maximum of the cosinor fit. It can be described as the timing of overall high values in the cycle, expressed in (negative) degrees, where the reference time is set to 0°, and a full period is 360°. The period T is the duration of one cycle, which is 24 h for circadian cycles.

### 2.4 Measurement of Serum Leptin Levels

To evaluate leptin levels, blood samples were taken at the end of the experiment, thus at the end of chronic starvation (day 29; Control_starvation: *n* = 11; SIH_starvation: *n* = 10) and refeeding phase (day 49; Control_refeeding: *n* = 11; SIH_refeeding: *n* = 10) via retro‐orbital blood draw, starting after nighttime and ending before the feeding time (7 a.m.–1 p.m.). Therefore, a glass capillary was carefully inserted into the inner corner of the eye until blood flowed. To determine serum leptin levels, blood was centrifuged at 6000 rpm for 15 min at 4°C after collection and serum was collected. Serum leptin levels were measured by performing a Mouse/Rat Leptin Quantikine ELISA (R&D Systems, Minneapolis, MN, USA) according to the manufacturer's instructions.

### 2.5 Reverse Transcription (RT) and Real‐Time Polymerase Chain Reaction (rtPCR)

At the end of the experiment (day 29: Control_chronic: *n* = 11, SIH_chronic: *n* = 10), mice were given intraperitoneal injections of ketamine (100 mg/kg) and xylazine (10 mg/kg). The perfusion times were kept as constant as possible (7 a.m.–1 p.m.). The brains were quickly dissected after transcardial perfusion with phosphate‐buffered saline (PBS) and hypothalamus containing SCN was prepared. Samples were immediately frozen in liquid nitrogen and stored at −80°C until analysis.

The mRNA of hypothalamus samples was isolated using RNA‐Solv Reagent (Omega Bio‐tek, Norcross, GA, USA). Accordingly, all samples were reverse transcribed in complementary DNA with M‐MLV RT‐kit (Invitrogen, Waltham, MA, USA). The circadian genes studied were *Bmal1*, *Rev‐erbα*, *Per1*, and *Cry1*. Glyceraldehyde‐3‐phosphate dehydrogenase (*Gapdh*) was used as a housekeeping gene as it is expressed at consistent levels in various tissues and treatment conditions. All primers used are listed in Table 1. All rtPCRs used SYBR Green technology (SensiMix SYBR & Fluorescein Kit, Bioline, Luckenwalde, Germany) and were performed on a CFX Connect Real‐Time PCR Detection System (Bio‐Rad Laboratories, Hercules, CA, USA). The relative expression was measured by calculating the ratio between the gene of interest and the housekeeping gene *Gapdh* by the ΔΔCt‐method (Livak and Schmittgen 2001) (further details: Data S1). Changes in gene levels of interest were graphically illustrated by the fold change relative to the control group. Due to insufficient RNA amount, some animals (Control_chronic: *n* = 3; SIH_chronic: *n* = 5) were excluded from the analyses.

**TABLE 1:** List of primers used for rtPCR.

Target gene	Sense	Oligonucleotides	Ta (°C)
*Gapdh*	Forward	ACAACTTTGGCATTGTGGAA	64
	Reverse	GATGCAGGGATGATGTTCTG	
*Bmal1*	Forward	CACCGTGCTAAGGATGGCTG	65
	Reverse	CTGCTGCCCTGAGAATTAGG	
*Rev‐erbα*	Forward	CCCTGGACTCCAATAACAACACA	62
	Reverse	GCCATTGGAGCTGTCACTGTAG	
*Per1*	Forward	CCAGATTGGTGGAGGTTACTGAGT	65
	Reverse	GCGAGAGTCTTCTTGGAGCAGTAG	
*Cry1*	Forward	GGAAGGAACGAGATGCAGCT	62
	Reverse	AGTGGCTCCATCTTGCTGAC	

### 2.6 Immunohistochemistry and Image Analysis

To evaluate cell densities after chronic starvation, animals included in our previous study were used (Control_chronic: *n* = 5; SIH_chronic: *n* = 10) (Staffeld et al. 2023). These animals were approved by the Review Boards for the Care of Animal Subjects of the district government of Mecklenburg‐Western Pomerania (reference number 7221.3‐1‐005/21). The animals were treated with the same parameters (25% weight reduction, early adolescent mice (four weeks old), and chronic starvation), leading to hyperactivity and amenorrhea during chronic starvation (Staffeld et al. 2023). At the end of the experiment, after nighttime (7 a.m.), mice were given intraperitoneal injections of ketamine (100 mg/kg) and xylazine (10 mg/kg). After transcardial perfusion with PBS and 3.7% paraformaldehyde solution (pH 7.4), the brains were rapidly dissected. Tissue preparation was performed following standard procedures, with brains frontally sectioned into 40 μm slices and every third slice mounted for Nissl staining (Staffeld et al. 2023; Zimmermann et al. 2023). As previously described, immunohistochemistry was conducted using standard protocols (Zimmermann et al. 2023) (further details: Data S1). The antibodies were diluted in 5% goat or rabbit serum (Sigma, Munich, Germany) as indicated in Table 2. Two stained sections per animal were digitized, and their results were averaged. The software QuPath Version 0.5.0 (Bankhead et al. 2017) was used to quantify GFAP^+^, ionized calcium‐binding adaptor molecule 1‐positive (IBA1^+^), and oligodendrocyte transcription factor 2‐positive (OLIG2^+^) cells. Cell counts were performed by two evaluators who were blinded to the treatment groups and expressed as cells per mm^2^. All GFAP^+^, IBA1^+^, and OLIG2^+^ cells with a visible nucleus were counted, excluding damaged sections. In terms of SCN localization, the sections between Bregma −0.34 and −0.82 mm were used. The SCN near the third ventricle was manually reconstructed for each section using the Allen Mouse Brain Atlas (Franklin and Paxinos 2005) and continuous Nissl staining of every third brain slice (Figure S2).

**TABLE 2:** List of antibodies used for immunohistochemical stains.

Antigen	Species	Dilution	Clonality	Purchase number	RRID	Supplier
Primary antibodies						
GFAP	Goat	1:500	Polyclonal	SAB2500462‐100UG	AB_10603437	Sigma‐Aldrich, Germany
IBA1	Rabbit	1:2000	Polyclonal	019–19741	AB_839504	Wako, Japan
OLIG2	Goat	1:500	Polyclonal	AF2418	AB_2157554	Bio‐Techne, USA
Secondary antibodies						
Anti‐goat IgG	Rabbit	1:250	Polyclonal	BA‐5000	AB_2336126	Vector Laboratories, USA
Anti‐rabbit IgG	Goat	1:250	Polyclonal	BA‐1000	AB_2313606	Vector Laboratories, USA

### 2.7 Statistics

The data are presented as means (averaged over all animals in a group) and standard errors of the mean (SEM). All data and statistics can be found in Table S1. First, all data sets were tested for normal distribution using the Shapiro–Wilk test. For statistical analysis, the values of running activity in the different periods of the day (FAA, post‐prandial activity, night activity and pre‐prandial activity) were compared within the different phases: acclimatization phase (days 1–10), acute starvation phase (days 11–16), chronic starvation phase (days 17–29) and refeeding phase (days 30–49). The analysis between SIH and control mice within each phase was performed using two‐way ANOVA with repeated measurements with a significance level of *p*‐values ≤ 0.05. Bonferroni's correction was used for post hoc evaluations between the control and SIH groups. Gene expression and cell density data were analyzed using Student's *t*‐test or, if the data were not normally distributed, the Mann–Whitney test. In addition, serum leptin levels were analyzed using two‐way ANOVA with Bonferroni's correction. *p*‐values ≤ 0.05 were considered statistically significant. These statistical analyses were conducted with SPSS version 20 (IBM, Chicago, IL, USA) and GraphPad Prism 10.2 (GraphPad Software, Boston, MA, USA).

Furthermore, the mixed‐effects cosinor model provided by the cosinoRmixedeffects package for R was used to model the longitudinal wheel activity data (Hou, Tomalin, and Suárez‐Fariñas 2022). The model included the hourly wheel activity as the dependent variable, with the independent factors being the phase of starvation (acclimatization phase: days 1–10; acute starvation phase: days 11–16; chronic starvation phase: days 17–29 and refeeding phase: days 30–49) and the grouping factor (Control_refeeding: *n* = 11; SIH_refeeding: *n* = 10). The interaction term between the phase of starvation and group (starvation phase × group) was also included. Random effects were assumed for MESOR, amplitude and acrophase for each animal. To estimate means and mean differences, a bootstrapping method with 500 simulations was used. The cosinor‐based analyses were conducted using R software version 4.3.2 (R foundation for Statistical Computing, Vienna, Austria 2021).

The sample size was determined using an a priori *one‐way* ANOVA power analysis with the software G*Power Version 3.1.4 (Faul et al. 2007) (further details: Data S1).

## 3 Results

### 3.1 Starvation‐Induced Increased Food‐Anticipatory Activity is Reversed by Refeeding

**FIGURE 2:**
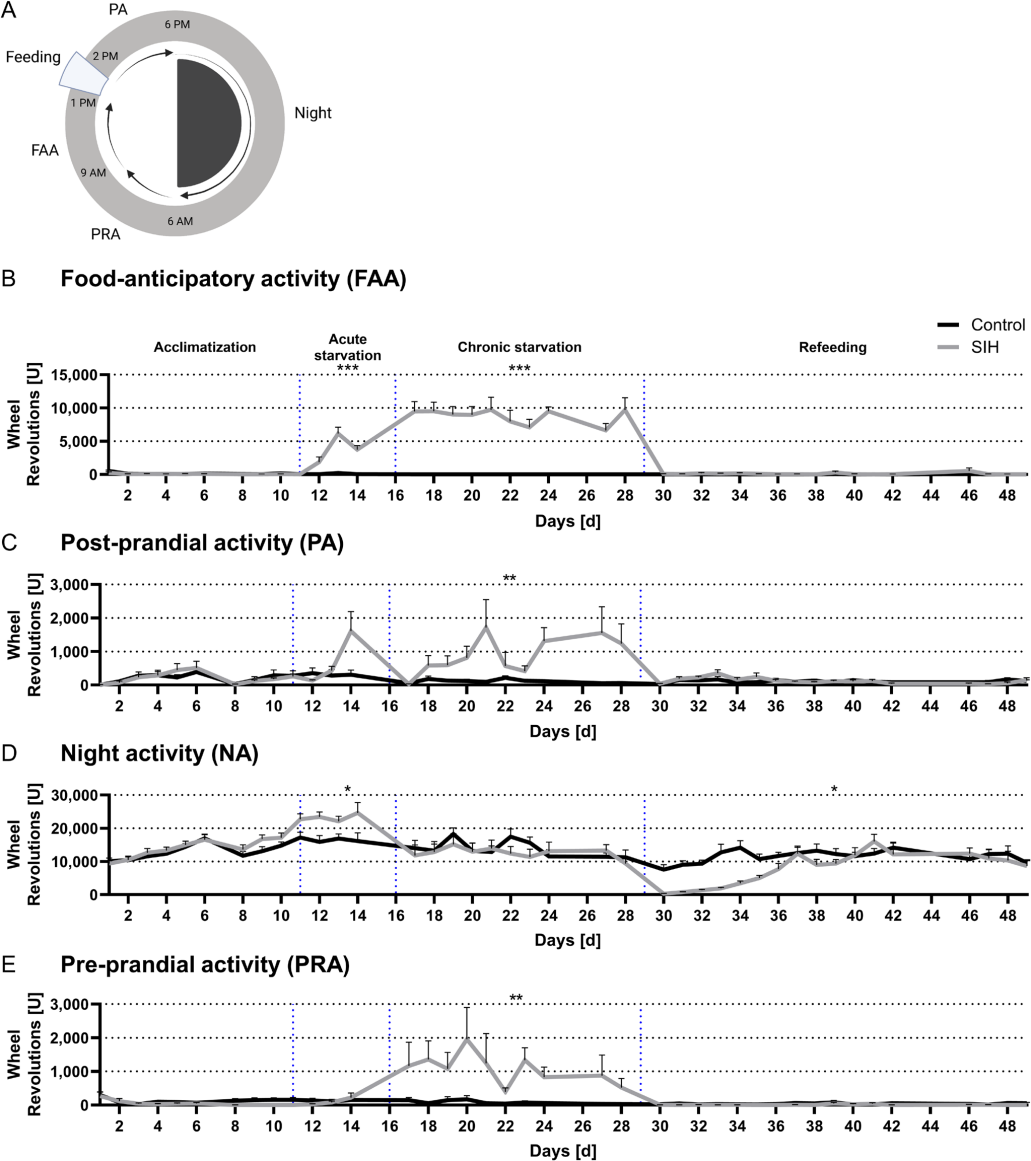
The increased food anticipatory activity associated with starvation is normalized with refeeding. (A) A 24 h cycle with the following phases was used as the experimental structure to analyze the different daily running activity periods. (B) The food anticipatory activity (FAA, 4 h, from 9 a.m. to 1 p.m.), (C) the post‐prandial activity (4 h, from 2 to 6 p.m.), (D) the night activity (12 h, 6 p.m. to 6 a.m. next day) and (E) the pre‐prandial activity (3 h, from 6 to 9 a.m.) stages during acute starvation, chronic starvation and refeeding were investigated using running wheel sensors. Two‐way ANOVA with repeated measurements, *p ≤ 0.05, **p ≤ 0.01, ***p ≤ 0.001 (Control_refeeding: *n* = 11; SIH_refeeding: *n* = 10).

To analyze the running wheel activity of the mice, 24 h cycles were divided into the following daily periods (Figure 2): food anticipatory activity (FAA, 4 h, from 9 a.m. to 1 p.m.), post‐prandial activity (4 h, from 2 to 6 p.m.), night activity (12 h, 6 p.m. to 6 a.m. next day) and pre‐prandial activity (3 h, from 6 to 9 a.m.).

During the phase of acclimatization, no differences in running activity were observed between SIH and control mice during any period of the day. Acute starvation led to an increase in FAA and remained increased in SIH mice during chronic starvation (Figure 2B). In addition, post‐prandial activity was also increased during chronic starvation compared to the control group (Figure 2C). While acute starvation led to an increase in night activity, there was no difference detectable in SIH mice compared to controls during chronic starvation (Figure 2D). During the refeeding phase, a significant reduction in night activity was measured (Figure 2D). Furthermore, pre‐prandial activity was increased during chronic starvation compared to controls (Figure 2E). No differences were observed in FAA, post‐prandial activity, and pre‐prandial activity during refeeding, indicating a normalized daily rhythm compared to controls. In summary, chronic starvation resulted in alterations in daily locomotor activities as indicated by increased FAA, post‐prandial activity, and pre‐prandial activity, while refeeding reversed these effects.

### 3.2 Starvation‐Induced Changes in Cosinor‐Based Rhythmicity are Reversed by Refeeding

**FIGURE 3:**
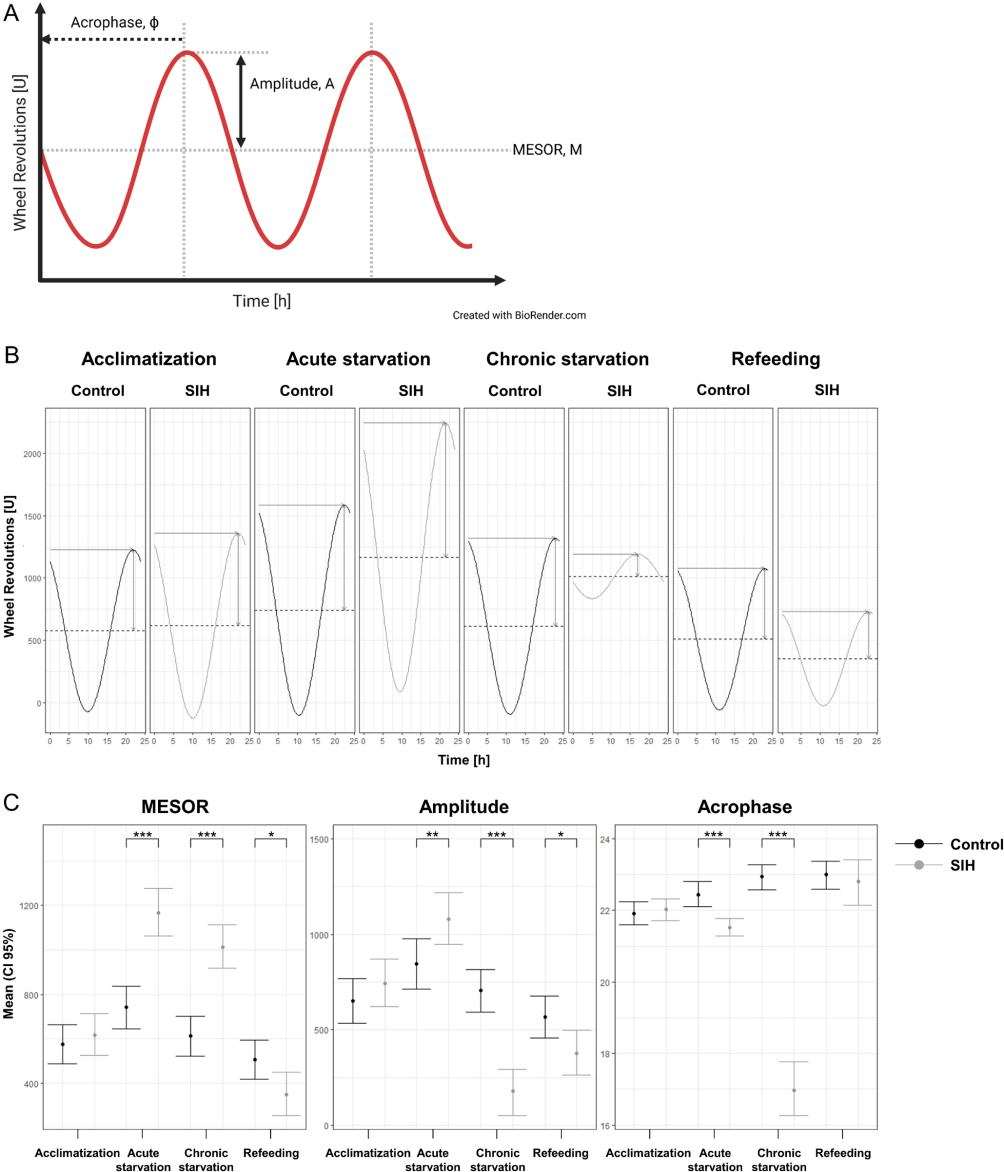
Starvation induces changes in cosinor‐based rhythmicity which are normalized by refeeding. (A) Circadian rhythm analyses used a cosine curve to fit running wheel activity data, with parameters including MESOR (average activity level), amplitude (amount of peak activity), and acrophase (timing of peak activity in degrees). (B) Curve fits generated from cosinor circadian rhythm analysis of running wheel activity. The dotted horizontal line represents mean activity (MESOR), the vertical arrow indicates the maximum extent of circadian activity (amplitude), and the horizontal arrow denotes the onset of the amplitude (acrophase). (C) Cosinor circadian rhythm analysis parameters of running wheel activity, including MESOR, amplitude and acrophase, are presented as estimated mean values with 95% confidence intervals. Bootstrapping statistics, *p ≤ 0.05, **p ≤ 0.01, ***p ≤ 0.001 (Control_refeeding: *n* = 11; SIH_refeeding: *n* = 10).

To investigate the rhythmicity of the circadian cycle, cosinor‐based analyses of running wheel activity were performed and the findings are depicted in Figure 3.

Mean activity, as indicated by MESOR, was higher during both acute and chronic starvation in SIH mice compared to control mice. Furthermore, an increase in the extent of rhythmic activity was observed in SIH mice, as reflected by a higher amplitude during the acute starvation phase. In contrast, there was a decrease in amplitude during the chronic starvation phase. Additionally, the peak activity time in the circadian cycle, indicated by amplitude, was shifted to earlier values during both acute and chronic starvation phases in SIH mice as compared to the control group. While MESOR normalized to a decreased level, the amplitude persisted at a slightly reduced level during refeeding. In contrast, there was no difference in acrophase during refeeding, indicating a normalized rhythm.

In summary, acute and chronic starvation led to changes in the circadian rhythm, as indicated by significantly altered cosinor characteristics of running wheel activity in SIH mice. During the refeeding phase, the circadian cycle was normalized compared to the control group.

### 3.3 Starvation‐Induced Decreased Serum Leptin Levels are Reversed by Refeeding

Next, it was investigated whether changes in circadian rhythm during starvation are related to changes in the endocrinological system, especially the satiety hormone leptin.

**FIGURE 4:**
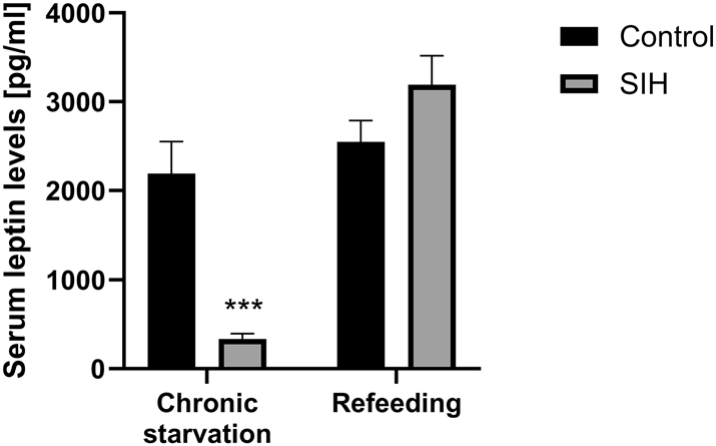
Refeeding after chronic starvation leads to a reversion of decreased serum leptin levels. All parameters from each mouse were determined at the end of the experiment. Two‐way ANOVA, ***p ≤ 0.001 (Control_chronic: *n* = 11; SIH_chronic: *n* = 10; Control_refeeding: *n* = 11; SIH_refeeding: *n* = 10).

After chronic starvation, serum leptin levels were reduced compared to controls (Figure 4). After refeeding, no change in leptin levels was observed in SIH mice compared to controls. To summarize, changes in circadian rhythm during chronic starvation in SIH mice are paralleled by a reduction in serum leptin levels, which also normalized after refeeding.

### 3.4 Starvation Induces an Increase in *Cry1* Expression in the Hypothalamus

Furthermore, we investigated whether circadian disruptions related to starvation also affect the mRNA expression of the clock genes *Bmal1*, *Rev‐erbα*, *Per1*, and *Cry1*.

**FIGURE 5:**
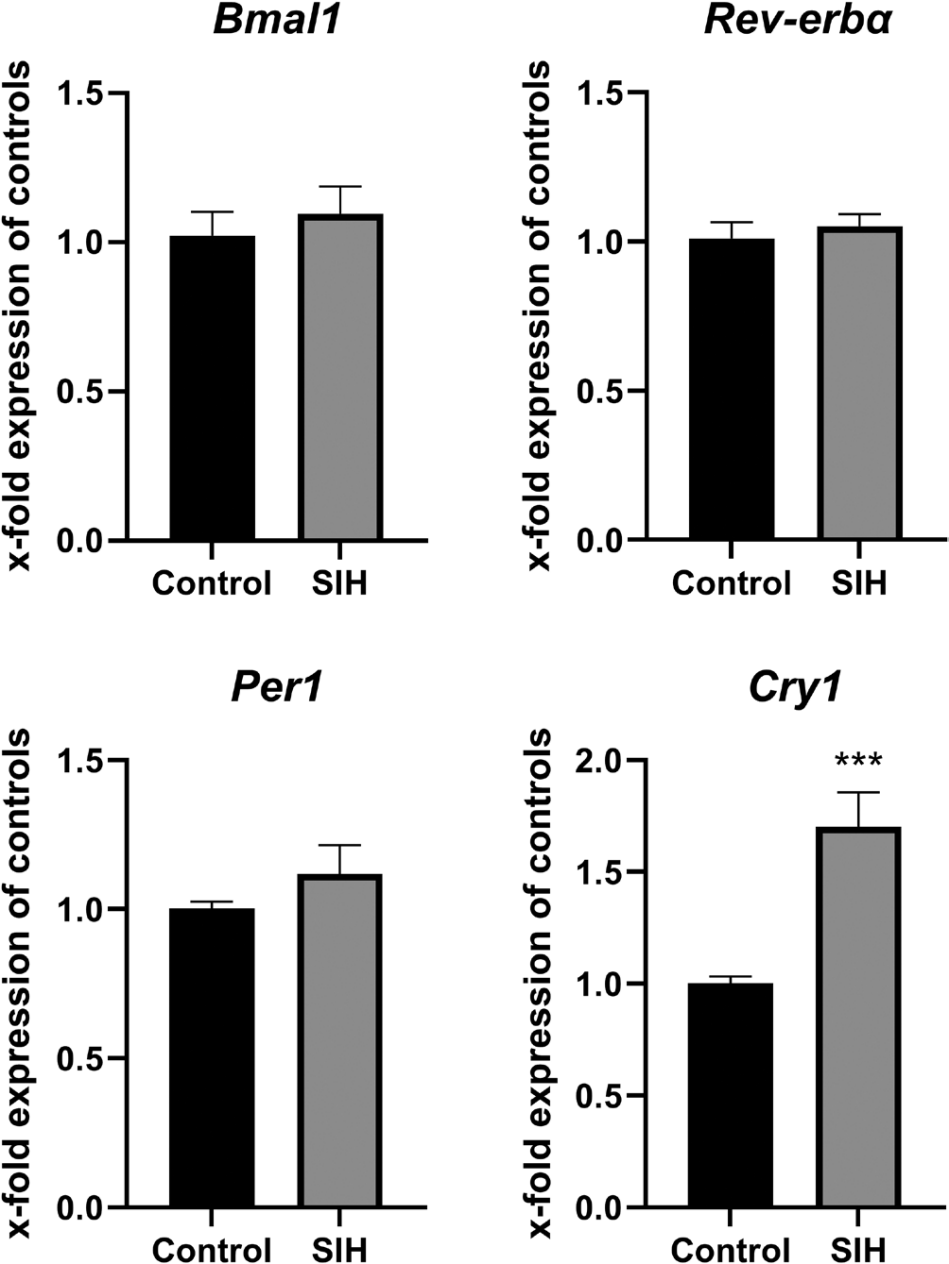
Starvation leads to an increased *Cry1* expression. *Bmal1*, *Rev‐erbα*, *Per1*, and *Cry1* mRNA expression in the hypothalamus of SIH and control mice after chronic starvation. Two‐sided Student's *t*‐test, ***p ≤ 0.001 (Control_chronic: *n* = 8; SIH_chronic: *n* = 5).

While there were no changes in *Bmal1*, *Rev‐erbα* and *Per1* mRNA expression in SIH mice compared to the control group, chronic starvation induced an increase in *Cry1* gene expression (Figure 5).

### 3.5 Starvation Induces a Reduction in GFAP ^+^ and OLIG2 ^+^ Cell Densities in the SCN

**FIGURE 6:**
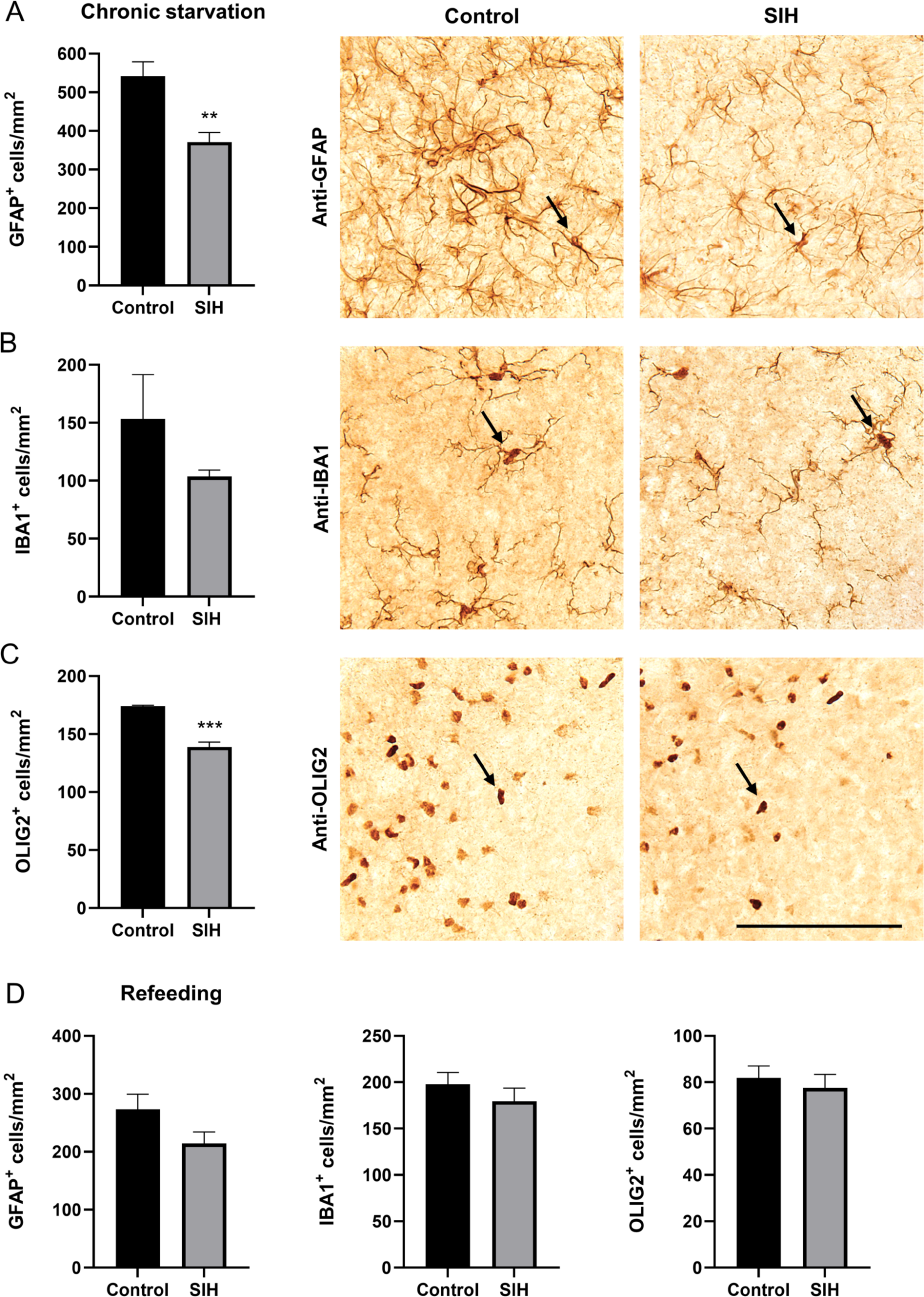
Chronic starvation leads to decreased astrocyte and oligodendrocyte cell density in the suprachiasmatic nucleus. After refeeding, glial cell densities in the suprachiasmatic nucleus remain unaltered. Cell densities of (A) GFAP^+^, (B) IBA1^+^, and (C) OLIG2^+^ in the suprachiasmatic nucleus of SIH and control mice after chronic starvation. The arrows mark (A) GFAP^+^, (B) IBA1^+^, and (C) OLIG2^+^ cells. Scale bar = 100 μm. Two‐sided Student's *t*‐test for GFAP^+^ and OLIG2^+^ cells, Mann–Whitney test for IBA1^+^ cells. (D) Cell densities of GFAP^+^, IBA1^+^, and OLIG2^+^ in the suprachiasmatic nucleus of SIH and control mice after refeeding. Two‐sided Student's *t*‐test. **p ≤ 0.01, ***p ≤ 0.001 (Control_chronic: *n* = 4; SIH_chronic: *n* = 8; Control_refeeding: *n* = 10; SIH_refeeding: *n* = 9).

Next, we analyzed the influence of starvation on glial cells in the SCN of the hypothalamus (Figure 6), as changes in circadian rhythm may be induced by cellular alterations in the SCN.

Chronic starvation induced a decrease in GFAP^+^ cells in this region (Figures 6A and S3). Additionally, although not significantly different, starvation led to a reduction in the density of IBA1^+^ microglial cells. Further, chronic starvation led to a reduction of OLIG2^+^ cells in SIH mice compared to controls (Figures 6C and S3). After refeeding, no alteration in glial cell densities was observed in SIH mice compared to controls (Figures 6D and S4). In summary, while chronic starvation led to reduced densities of GFAP^+^ and OLIG2^+^ cells in the SCN, the density of IBA1^+^ cells remained unchanged.

## 4 Discussion

AN is a severe psychiatric disorder characterized by extreme weight loss and distorted body image (Herpertz‐Dahlmann 2015). It is often associated with extensive locomotor activity and circadian disruptions (Herpertz‐Dahlmann 2015; Allison, Spaeth, and Hopkins 2016; Wilcox et al. 2024).

First, we investigated whether starvation in a mouse model leads to changes in FAA, post‐prandial activity, night activity and pre‐prandial activity. SIH mice demonstrated increased food‐anticipatory behavior after acute and chronic starvation, consistent with our previous study (Gabloffsky et al. 2022), which can be related to food‐seeking behavior or anticipation of feeding (Adan et al. 2011). The FAA is regulated by hypothalamic neuronal circuits, particularly by the brain's master clock, the SCN (Merkestein, Verhagen, and Adan 2013). Chronobiologists assume the presence of a second pacemaker, the food‐entrained oscillator (FEO), located outside the SCN (Mistlberger 2009). Circadian rhythm‐related FAA was observed in SCN‐ablated rats, suggesting that the FEO driving anticipatory activity may be independent of the SCN (Marchant and Mistlberger 1997; Boulos, Rosenwasser, and Terman 1980; Yoshihara et al. 1997). More precisely, the regulation of FAA is probably distributed across a network of brain nuclei (Merkestein, Verhagen, and Adan 2013). For example, a study in an AN rat model showed an involvement of the arcuate nucleus (ARC) in the hypothalamus in the anticipation of food delivery (Verhagen et al. 2011). This is consistent with a study that suggested that Agouti‐related protein (AgRP)‐expressing neurons in the ARC integrate information about the time of day from past feeding experiences with current metabolic needs to predict circadian feeding time (Sayar‐Atasoy et al. 2024). Moreover, FEOs might be located in other additional brain regions, including dopamine D1 receptor‐expressing neurons in the dorsal striatum (Gallardo et al. 2014) and the medial prefrontal cortex‐dorsal striatum pathway (Santiago et al. 2021). In addition, refeeding reversed the increased FAA, indicating a normalized daily rhythm compared to controls, which is in line with another study investigating running wheel activity in SIH mice (Du Tezenas Montcel et al. 2023).

Furthermore, we observed other changes in daily activities suggesting that a change in FAA may affect other periods of the day. Post‐prandial activity and pre‐prandial activity increased during chronic starvation, suggesting that changes in daily locomotor activities are not limited to food‐seeking behavior immediately before feeding and that additional mechanisms may be involved. One study showed that post‐prandial activities in SIH mice were associated with weight loss, suggesting that FAA may not be sufficient to reflect the severity of the model (Wu et al. 2014). Moreover, since mice are nocturnal animals, they exhibit increased activity during the night, as confirmed by the high number of revolutions during the dark period. Refeeding reversed the increased post‐prandial activity and pre‐prandial activity in SIH mice, indicating a normalization of the daily rhythm compared to controls, which is consistent with the findings of the FAA in SIH mice. Furthermore, the initial decrease in running wheel activity during nighttime was probably caused by an increase in food consumption immediately after refeeding, as shown in a previous study (Trinh et al. 2023). In future studies, the effects of an increase in FAA on daily locomotor activities and circadian rhythm will be more precisely investigated by shifting the feeding time to the nighttime.

The cosinor‐based rhythmicity analysis demonstrated that chronic starvation leads to a change in circadian rhythmicity, as indicated by altered MESOR, amplitude, and acrophase of running wheel activity in SIH mice. Compared to the analysis of daily locomotor activities, the cosinor‐based analysis provides an insight into circadian rhythms by fitting a cosine curve to periodic data. Therefore, cosinor‐based characteristics offer quantitative evidence that not only daily locomotor activities are altered. To the best of our knowledge, for the first time an altered MESOR, amplitude, and acrophase of running wheel activity in SIH mice were demonstrated, indicating that the circadian rhythm of SIH mice is altered. As previously shown in daily locomotor activities, cosinor‐based rhythmicity also supports the finding that refeeding normalized the daily activity rhythm as indicated by normalized cosinor‐based characteristics.

Next, we investigated whether changes in circadian rhythm after chronic starvation are associated with changes in leptin levels. Clock genes are known to modulate leptin and its signaling pathways by suppressing the expression of inhibitors of leptin signaling, thereby activating the leptin signaling pathway and increasing leptin sensitivity (Ansarin et al. 2023; Adlanmerini et al. 2021; Kaneko et al. 2009). Conversely, leptin influences the expression of clock genes by initiating signaling cascades (Ansarin et al. 2023). Consistent with previous studies (Rijke et al. 2005; Verhagen et al. 2009), our study demonstrated that chronic starvation leads to decreased leptin levels. In addition, studies have demonstrated an altered circadian pattern of leptin secretion in patients with AN (Herpertz et al. 1998), which may be associated with altered circadian rhythms in patients with AN (Allison, Spaeth, and Hopkins 2016). There are reports that in patients with AN treated with human recombinant leptin (metreleptin) increasing leptin levels are associated with improved sleep quality (Hebebrand, Denecke, and Antel 2023; Antel et al. 2022; Gradl‐Dietsch et al. 2023; Milos et al. 2020). This may indicate that circadian rhythm changes are caused by leptin deficiency. Furthermore, continuous treatment with leptin could reduce hyperactivity in SIH rats (Exner et al. 2000). Also, FAA was reduced during starvation in SIH rats treated with leptin compared to SIH rats treated with vehicle (Verhagen et al. 2009). These findings are in line with the observation that FAA was reversible by refeeding, due to the normalized leptin levels. Therefore, leptin deficiency may contribute to the development of the observed FAA.

In addition, we investigated whether changes in the circadian rhythm in SIH mice were associated with changes at the molecular level, as indicated by the expression of clock genes in the hypothalamus. Clock genes regulate circadian rhythms by promoting the positive feedback loop (*Bmal1*) or the negative feedback loop (*Rev‐erbα*, *Per1*, *Cry1*), thereby activating or inhibiting the transcription of *Per1* and *Cry1* (reviewed in (Cox and Takahashi 2019)). Our study revealed that the *Cry1* gene expression in the hypothalamus was increased after starvation compared to controls. In another AN mouse model, the *Bmal1* gene expression was found to be decreased in the hypothalamus (Salaün et al. 2024). Nevertheless, both findings suggest an imbalance in the expression of clock genes, potentially triggering the negative feedback loop, which could contribute to changes in circadian rhythms in SIH mice. In terms of that, it should be noted that the analysis of clock genes was not performed on the SCN exclusively, but rather on the entire hypothalamus. However, Drunen et al. revealed that circadian clock gene expression also occurs in other hypothalamic nuclei, such as the paraventricular nucleus (van Drunen et al. 2024). This suggests that circadian disruptions may also underlie systemic changes in clock gene expression, rather than isolated gene expression changes in the hypothalamus.

In terms of potential changes in glial cells that could be involved in circadian disruption, we have shown a decreased density of astrocytes in the SCN after chronic starvation in the SIH mice. We assume that changes in circadian rhythm are associated with the observed decrease in astrocyte density. This is in line with studies indicating that astrocytes are essential components of the SCN oscillator, playing a significant role in circadian clock function (Costa and Montagnese 2021). Moreover, we previously showed that a reduction in astrocyte density in SIH animals may contribute to the pathophysiology of AN, as seen in the cerebral cortex and corpus callosum (Frintrop, Liesbrock, et al. 2018; Frintrop et al. 2019; Zimmermann et al. 2023). In addition, no alteration in astrocyte density reduction was observed in the ARC after chronic starvation (Zimmermann et al. 2023). Although not significantly changed, starvation led to a reduced density of IBA1^+^ cells in the SCN. This is in line with our previous study in SIH mice which showed a reduction in IBA1^+^ cells in the corpus callosum (Zimmermann et al. 2023). In contrast, studies in DIA rats showed increased IBA1^+^ cell density in the prefrontal cortex and hippocampus (Reyes‐Haro et al. 2015; Ragu‐Varman et al. 2019). Therefore, depending on the type of starvation and the specific brain region affected, microglial cells may respond differently. In addition, we showed that OLIG2^+^ cells were significantly reduced in the SCN after chronic starvation, which is consistent with our previous findings that OLIG2^+^ cells were reduced in the corpus callosum in SIH mice (Zimmermann et al. 2023). Taken together, this suggests that glial cell dysfunction rather than microgliosis or inflammation contributes to the circadian rhythm during starvation. Additionally, glial cells, especially astrocytes, play an important role in regulating synaptic plasticity through various mechanisms, including the secretion of neuroactive substances and the removal of neurotransmitters (Paixão and Klein 2010). Therefore, astrocyte dysfunction due to decreased cell density may lead to behavioral changes, such as the observed impairment in recognition memory (Paulukat et al. 2016).

A limitation of the SIH model is the need for social isolation of the mice, which may cause stress. This factor is counterbalanced, as control mice were housed under the same conditions. Future studies should include a control group without running wheel access and a food‐restricted group without a wheel to distinguish starvation‐induced effects of activity from food restriction.

In summary, chronic starvation induced changes in circadian rhythm, and refeeding normalized this effect. Refeeding also reversed decreased serum leptin levels. Furthermore, *Cry1* expression was increased and GFAP^+^ and OLIG2^+^ cells were decreased in the SCN after chronic starvation. This suggests that circadian disruptions in SIH mice involve several mechanisms and may be mediated by the hypothalamus. Nevertheless, further research should include additional daily time points of sample collection in order to gain a deeper insight into the mechanisms that lead to circadian disruptions.

To conclude, changes in circadian rhythm seem to be associated with changes at multiple levels in the hypothalamus, including endocrinological, molecular and cellular, in particular involving reduced astrocytes. As glial cell alterations seem to play an important role in the mechanisms underlying circadian disruption, the SIH model allows us to investigate their influence further and identify new therapeutic options.

## Author Contributions

**Annelie Zimmermann:** conceptualization, data curation, formal analysis, investigation, methodology, validation, visualization, writing – original draft, writing – review and editing. **Julia Priebe:** data curation, formal analysis, investigation, methodology, visualization. **Hanna Rupprecht:** investigation. **Stephan Lang:** investigation. **Fabienne Haberland:** formal analysis. **Katharina Schuster:** investigation. **Anna Staffeld:** investigation. **Christoph Berger:** data curation, formal analysis, validation, visualization. **Hang Zhu:** formal analysis. **Alexander Dück:** conceptualization, methodology, validation. **Michael Kölch:** resources, validation. **Linda Frintrop:** conceptualization, data curation, formal analysis, funding acquisition, investigation, methodology, project administration, resources, supervision, validation, visualization, writing – original draft, writing – review and editing.

## Ethics Statement

All animal procedures were performed at the Institute of Anatomy at the Rostock University Medical Center and carried out following the EU Directive 2010/63 for the protection of animals used for scientific purposes and the recommendations of the Federation of European Laboratory Animal Science Associations (FELASA). The animal studies were given approval by the Review Boards for the Care of Animal Subjects of the district government of Mecklenburg‐Western Pomerania (reference number 7221.3‐1‐005/21). The experiments have been reported following the ARRIVE (Animal Research: Reporting of In Vivo Experiments) guidelines (Du Percie Sert et al. 2020).

## Conflicts of Interest

The authors declare no conflicts of interest.

## Supporting information


**Data S1:** Supporting Information.

## Data Availability

Data may be made available upon sending requests to corresponding author.
