# Supplementary material for: Changes in Circadian Rhythm in Chronically‐Starved Mice Are Associated With Glial Cell Density Reduction in the Suprachiasmatic Nucleus
Source: Int J Eat Disord. 2025 Jan 21;58(4):756–69. doi: 10.1002/eat.24379 (PMC11969035; doi:10.1002/eat.24379)
Supplement: Supplementary file 1 — Data S1: Supporting Information. [file EAT-58-756-s001.pdf]

2.2 Study Design

Running wheel activity was analyzed using running wheels (11.5 cm in diameter) attached to the top of the cages, with revolutions recorded hourly by an activity program (VitalView Activity 1.4, STARR Life Science Corp.). Running wheel activity was assessed daily. The periods of a day were defined as follows: food anticipatory activity (FAA, 4 h, from 9 AM to 1 PM), post-prandial activity (4 h, from 2 PM to 6 PM), night activity (12 h, 6 PM to 6 AM next day) and pre-prandial activity (3 h, from 6 AM to 9 AM).

2.5 Real-time polymerase chain reaction

The residual volumes of all complementary DNA samples were combined to create a pooled standard. A dilution series of the standard (100%, 50%, 25%, 12.5%, 6.25%, 3.125%, 1.5625%) was prepared to evaluate whether a reliable amount of genetic material was present in the samples. Samples with Ct values lower than those of the most diluted standard were excluded from further analysis, ensuring reliability in the data. Following a linear regression on the data points from the standard series, the Bio-Rad CFX Maestro 1.0 software calculated a primer efficiency (= E) to ensure accurate quantification of relative gene expression.

The data were analyzed using the ΔΔCt method. Initially, the Ct values from duplicate measurements were averaged. Subsequently, the Ct values of the housekeeping gene were subtracted from those of the gene of interest to calculate the ΔCt values.

ΔCt = Ct (gene of interest) – Ct (housekeeping gene)

The mean ΔCt value of the control group was then subtracted from the ΔCt values of the chronic starvation group, yielding the ΔΔCt values.

ΔΔCt = ΔCt (treated sample) – ΔCt (untreated sample)

These values were incorporated into the equation for relative expression:

n-fold expression (chronic starvation group vs. control group) = $2^{-\Delta\Delta Ct}$.

If the efficiency was not exactly 100% (E = 2), the efficiency was included in the calculation (relative expression = $E^{-\Delta\Delta Ct}$).

2.6 Immunohistochemistry

Each brain was sectioned frontally into 40 µm slices using a cryostat (Leica CM 3050S, Nussloch, Germany), with every third section thaw-mounted on glass slides for Nissl staining according to standard protocols. The remaining sections were stored in a cryoprotectant solution at -20 °C for use in immunohistochemistry.

For immunohistochemistry, sections were treated with 5% goat or rabbit serum (Sigma, Munich, Germany) for 90 min and incubated overnight at 4 °C with the primary antibodies. Following this, sections were exposed to 0.35% hydrogen peroxide (H_2O_2) in PBS for 30 min and then to the corresponding secondary antibodies. This was followed by the ABC complex (Vector Laboratories, Burlingame, CA, USA). Antigenic sites were detected using 3,3’-diaminobenzidine (Dako, Hamburg, Germany). Negative controls (without primary antibodies) were used. The slides were digitalized with the Ocus20 microscope slide scanner (Grundium, Tampere, Finland; 20-fold objective, numerical aperture (NA): 0.40).

2.7 Statistics

The primary variable used to determine sample size was the brain atrophy, specifically the cerebral cortex volume [1,2]. Following starvation, the SIH animals showed a 9% reduction in cortical volume compared to the control group (SIH: 188.98 mm³, SD: 5.07; control: 206.51 mm³, SD: 3.61), resulting in a Cohen's d effect size of 1.24, and a Cohen's f of 0.62 [3]. Based on the G-Power software, 9 animals per group were required, but two additional animals were included to account for potential dropouts resulting in a total of 11 animals per group, consistent with our previous study.

Supplementary Table 1

A) Running wheel activity

**Food-anticipatory activity**					
Phase	Group	Mean	SEM	p-value	Cohen's d
Acute starvation	Control	79.14 U	41.73	≤ 0.001	1.58
	SIH	2933 U	1309		
Chronic starvation	Control	5.26 U	0.69	≤ 0.001	11.19
	SIH	8755 U	359.1		

**Post-prandial activity**					
Phase	Group	Mean	SEM	p-value	Cohen's d
Chronic starvation	Control	114.3 U	16.97	≤ 0.01	2.03
	SIH	881.3 U	172.8		

**Night activity**					
Phase	Group	Mean	SEM	p-value	Cohen's d
Acute starvation	Control	16544 U	309.4	≤ 0.05	7.59
	SIH	23207 U	548.7		
Refeeding	Control	11542 U	444.4	≤ 0.05	-1.03
	SIH	7842 U	1171		

**Pre-prandial activity**					
Phase	Group	Mean	SEM	p-value	Cohen's d
Chronic starvation	Control	79.80 U	16.76	≤ 0.01	3.19
	SIH	1074 U	142.3		

B) Cosinor-based rhythmicity

**MESOR**					
Phase	Group	Mean	SEM	p-value	Cohen's d
Acute starvation	Control	741.92	75.63	≤ 0.001	1.48
	SIH	1166.35	101.75		
Chronic starvation	Control	612.49	85.93	≤ 0.001	1.07
	SIH	1014.31	143.68		
Refeeding	Control	508.56	69.92	≤ 0.05	-0.60
	SIH	351.24	93.36		

Amplitude					
Phase	Group	Mean	SEM	p-value	Cohen's d
Acute starvation	Control	844.72	111.99	≤ 0.01	0.63
	SIH	1080.52	118.69		
Chronic starvation	Control	706.33	125.43	≤ 0.001	-1.34
	SIH	179.49	115.94		
Refeeding	Control	567.94	100.75	≤ 0.05	-0.51
	SIH	376.96	132.77		

Acrophase					
Phase	Group	Mean	SEM	p-value	Cohen's d
Acute starvation	Control	22.44	1.93	≤ 0.001	-0.18
	SIH	21.52	1.11		
Chronic starvation	Control	22.94	2.56	≤ 0.001	-0.90
	SIH	16.97	1.11		

C) Serum leptin levels

Phase	Group	Mean	SEM	p-value	Cohen's d
Chronic starvation	Control	2191.0 pg/ml	361.0	≤ 0.001	-2.11
	SIH	334.4 pg/ml	60.34		

D) *Cry1* mRNA expression

Phase	Group	Mean	SEM	p-value	Cohen's d
Chronic starvation	Control	1.00	0.03	≤ 0.001	3.20
	SIH	1.70	0.15		

E) Immunohistochemical stainings

GFAP					
Phase	Group	Mean	SEM	p-value	Cohen's d
Chronic starvation	Control	541.9 cells/mm^2	36.79	≤ 0.01	-2.40
	SIH	371.1 cells/mm^2	24.89		

OLIG2					
Phase	Group	Mean	SEM	p-value	Cohen's d
Chronic starvation	Control	174.1 cells/mm^2	0.78	≤ 0.001	-3.95
	SIH	138.9 cells/mm^2	4.11		

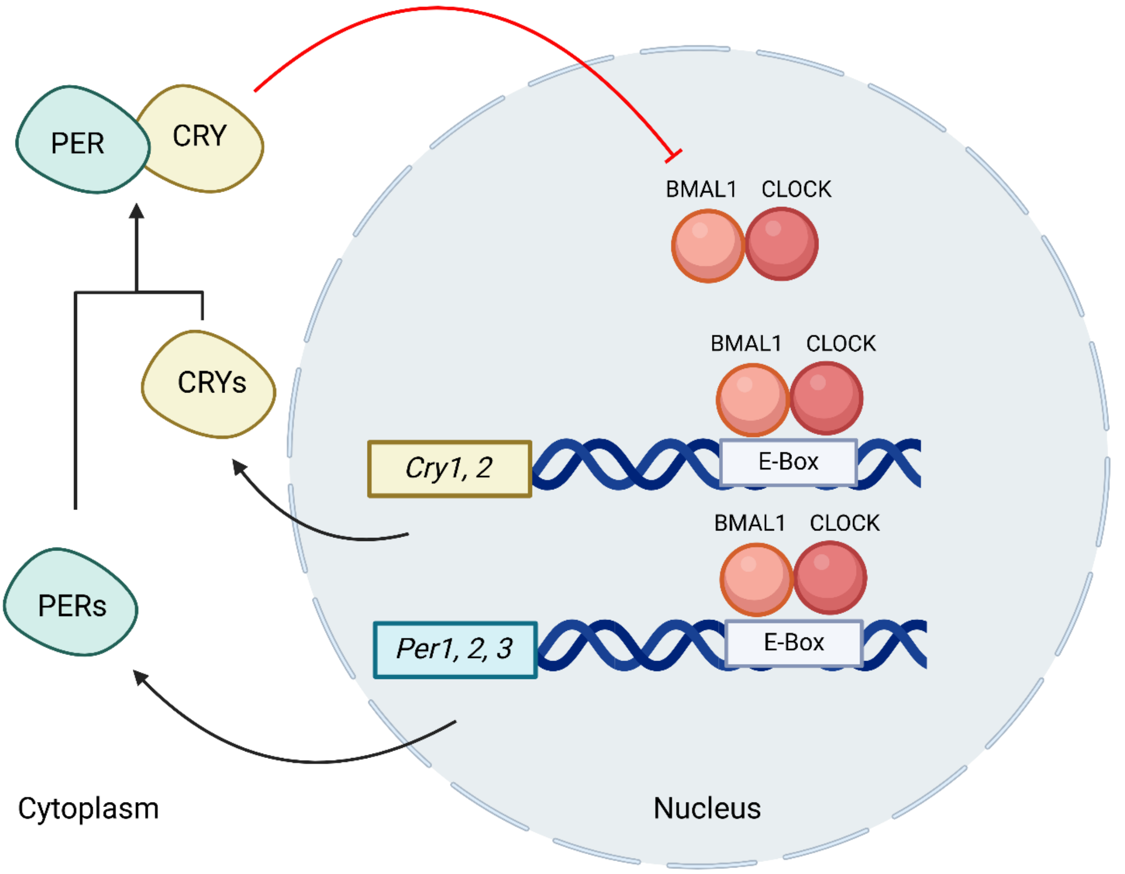

Figure S1: Protein circadian locomotor output cycles kaput (CLOCK) and brain and muscle ARNT-like 1 (BMAL1) bind directly to regulatory elements (E-boxes) on the Period (*Per1*, *Per2*, and *Per3*) and Cryptochrome (*Cry1*, *Cry2*) genes, thereby influencing their transcription. The PER and CRY complexes negatively regulate the BMAL1 and CLOCK proteins, thereby inhibiting their transcription. Created with BioRender.com.

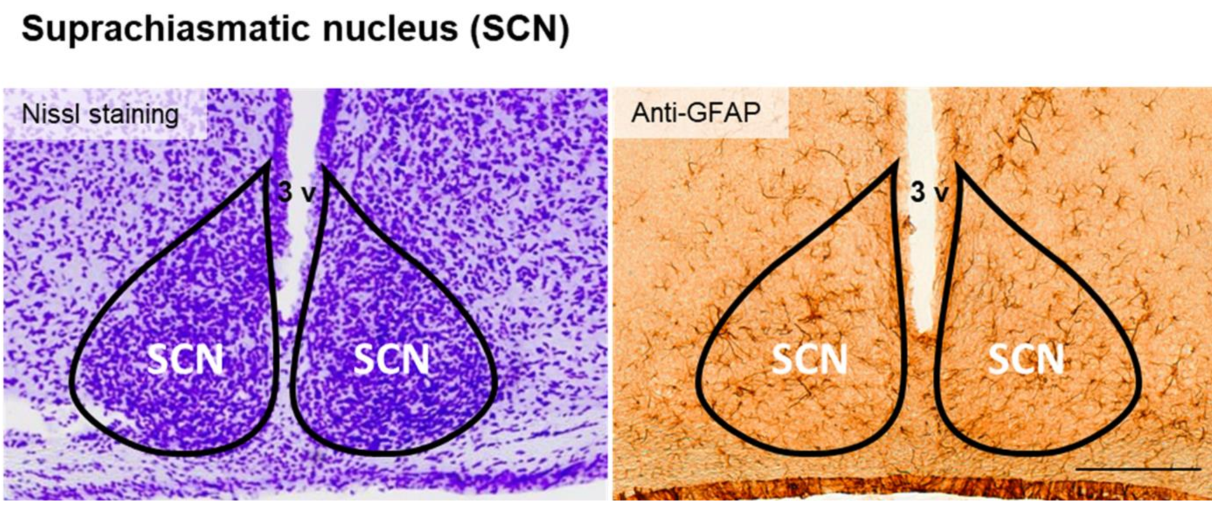

Figure S2: The Allen Mouse Brain Atlas and continuous Nissl staining of every third brain slice were utilized to reconstruct the region in the images of the immunohistochemical stainings for the suprachiasmatic nucleus (SCN), which is located near the third ventricle (3 v). Scale bar = 200 µm.

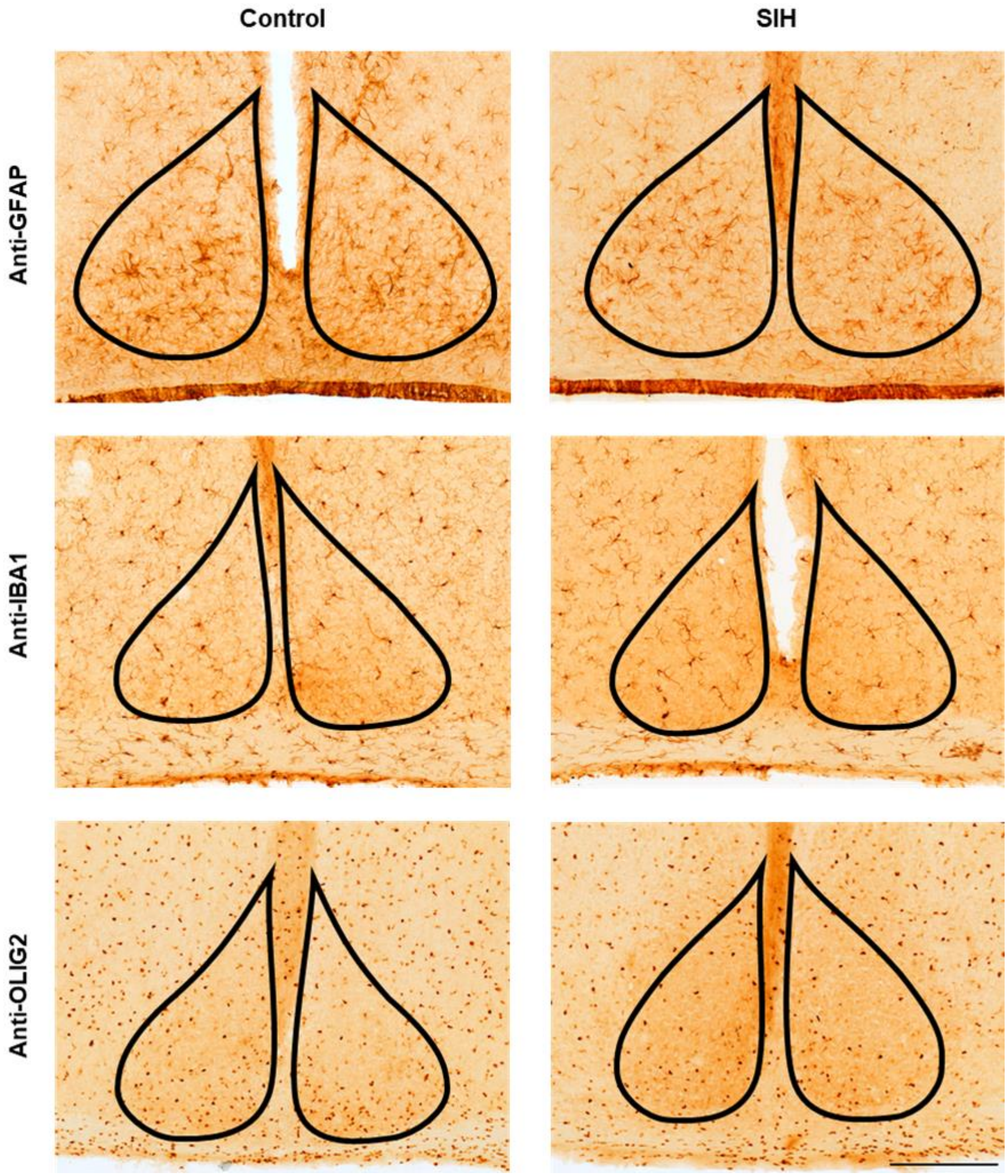

Figure S3: Immunohistochemical stainings of glial cells in the reconstructed suprachiasmatic nucleus with the lower part of the 3rd ventricle of SIH and control mice after chronic starvation. Scale bar = 200 µm.

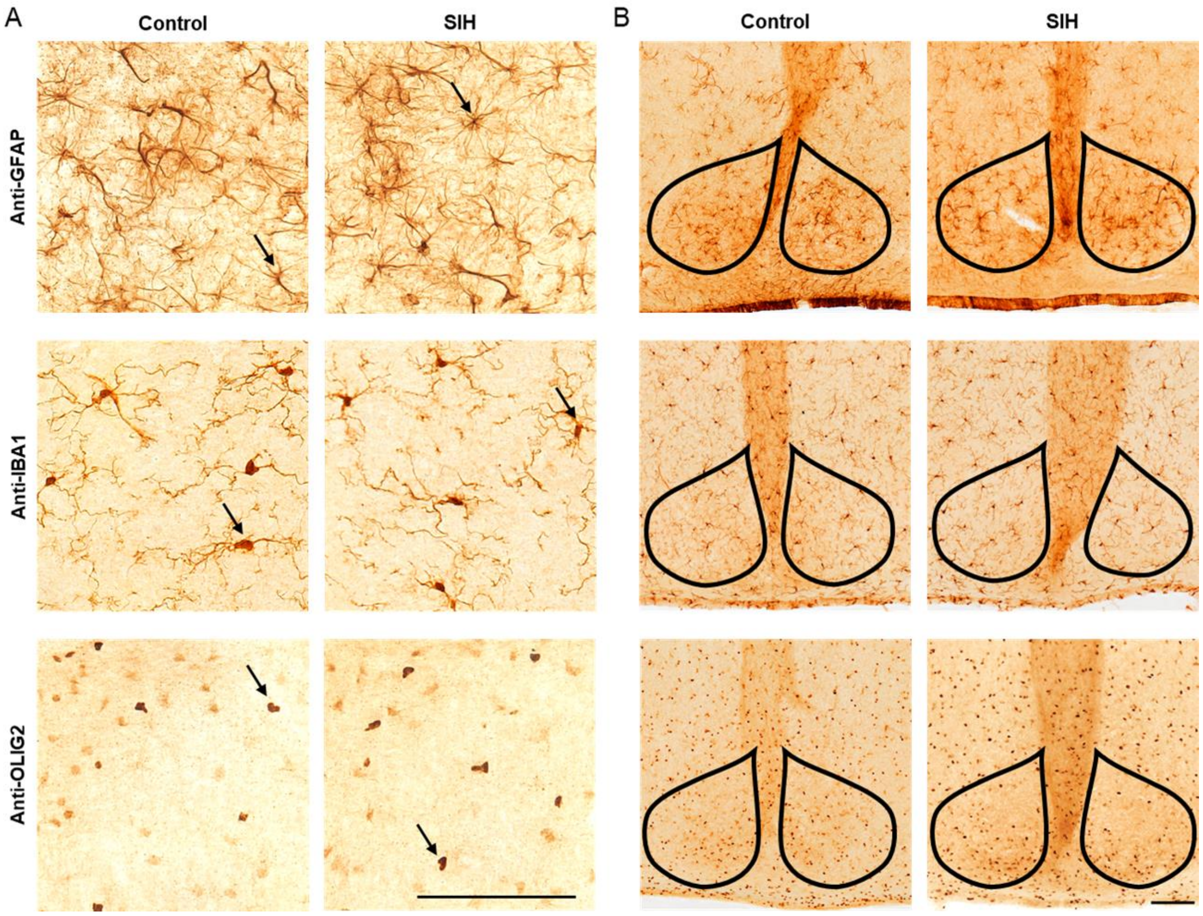

Figure S4: Immunohistochemical stainings of glial cells in the suprachiasmatic nucleus of SIH and control mice after refeeding. (A) The arrows mark GFAP⁺, IBA1⁺, and OLIG2⁺ cells. (B) The suprachiasmatic nucleus reconstructed with the lower part of the 3rd ventricle. Scale bar = 100 µm.

References

1. Frintrop, L.; Trinh, S.; Liesbrock, J.; Leunissen, C.; Kempermann, J.; Etdöger, S.; Kas, M.J.; Tolba, R.; Heussen, N.; Neulen, J.; et al. The reduction of astrocytes and brain volume loss in anorexia nervosa-the impact of starvation and refeeding in a rodent model. *Transl. Psychiatry* **2019**, *9*, 159, doi:10.1038/s41398-019-0493-7.
2. Staffeld, A.; Gill, S.; Zimmermann, A.; Böge, N.; Schuster, K.; Lang, S.; Kipp, M.; Palme, R.; Frintrop, L. Establishment of a Murine Chronic Anorexia Nervosa Model. *Cells* **2023**, *12*, doi:10.3390/cells12131710.
3. Wolfgang Lenhard; Alexandra Lenhard. *Computation of Effect Sizes*, 2017.
